# Comparing chronic condition rates using ICD-9 and ICD-10 in VA patients FY2014–2016

**DOI:** 10.1186/s12913-017-2504-9

**Published:** 2017-08-17

**Authors:** Jean Yoon, Adam Chow

**Affiliations:** 1 Health Economics Resource Center, VA Palo Alto, 795 Willow Rd, 152 MPD, Menlo Park, CA 94025 USA; 2 Center for Innovation to Implementation, VA Palo Alto, Menlo Park, CA USA; 3 UC San Francisco School of Medicine, San Francisco, CA USA

**Keywords:** Chronic disease, Diagnosis

## Abstract

**Background:**

Management of patients with chronic conditions relies on accurate measurement. It is unknown how transition to the ICD-10 coding system affected reporting of chronic condition rates over time. We measured chronic condition rates 2 years before and 1 year after the transition to ICD-10 to examine changes in prevalence rates and potential measurement issues in the Veterans Affairs (VA) health care system.

**Methods:**

We developed definitions for 34 chronic conditions using ICD-9 and ICD-10 codes and compared the prevalence rates of these conditions from FY2014 to 2016 in a 20% random sample (1.0 million) of all VA patients. In each year we estimated the total number of patients diagnosed with the conditions. We regressed each condition on an indicator of ICD-10 (versus ICD-9) measurement to obtain the odds ratio associated with ICD-10.

**Results:**

Condition prevalence estimates were similar for most conditions before and after ICD-10 transition. We found significant changes in a few exceptions. Alzheimer’s disease and spinal cord injury had more than twice the odds of being measured with ICD-10 compared to ICD-9. HIV/AIDS had one-third the odds, and arthritis had half the odds of being measured with ICD-10. Alcohol dependence and tobacco/nicotine dependence had half the odds of being measured in ICD-10.

**Conclusion:**

Many chronic condition rates were consistent from FY14–16, and there did not appear to be widespread undercoding of conditions after ICD-10 transition. It is unknown whether increased sensitivity or undercoding led to decreases in mental health conditions.

## Background

On October 1, 2015 U.S. health care providers covered under the Health Insurance Portability and Accountability Act (HIPAA) were required to transition from coding diagnoses during health care encounters using the International Classification of Diseases and Related Health Problems, 9th revision, Clinical Modification (ICD-9-CM) to the 10th revision (ICD-10-CM). ICD-10-CM uses 69,823 codes which is almost a five-fold increase in codes from ICD-9-CM. Measuring diagnoses accurately is critical to both patient and population management, so greater detail in ICD-10 improves upon the ICD-9 system. However, the transition to a new coding system poses challenges to measuring rates of chronic conditions over time within a health care system or population of interest. At least a third of ICD-9-CM codes do not have an easily discernible corresponding ICD-10 code, and some clinical specialties are affected more than others [1]. The implications of challenges in consistent coding are not yet understood although researchers comparing cause of death in ICD-9 and ICD-10 found significant differences that could be attributed to changes in coding in ICD-10 [2]. No current studies exist on how measuring chronic conditions in a population were affected by this transition.

We developed ICD-9 and ICD-10 definitions for 34 different chronic conditions, and we compared the rates of these conditions from fiscal years (FY) 2014 to 2016 in a large sample of VA patients. We compared the population rate of each condition in each study year. We also estimated how many patients with each condition in the current year did or did not have the condition diagnosed in the previous year, and how many who were previously diagnosed with the condition were not diagnosed in the current year to assess whether ICD-10 coding was associated with broader or more narrowly defined populations for each condition. Looking at how chronic condition rates changed before and after the transition to ICD-10 can help inform providers, clinical program managers, and researchers about measurement issues related to the transition to ICD-10.

## Methods

### Chronic condition codes

We created definitions for 34 common chronic conditions among VA patients for both ICD-9 and ICD-10 systems. Chronic conditions and corresponding ICD-9 diagnoses were identified from previous work and were selected because they were common and accounted for most of VA health care costs [3, 4]. For arthritis, asthma, coronary heart disease, diabetes, hypertension, stroke, heart failure, peripheral vascular disease, diabetes, renal disease, AIDS/HIV, we applied ICD-10 codes developed for comorbidity indices for the Charlson Index and Elixhauser Score [5, 6]. We used ICD-10 codes for spinal cord injury based on the definition developed by the Centers for Medicare & Medicaid Services (CMS) [7]. Several VA clinical workgroups developed comprehensive ICD-10 diagnosis lists for several chronic conditions, and we obtained the ICD-10 codes defined for chronic obstructive pulmonary disease, dementia, hepatitis C, ischemic heart disease, and lung cancer. ICD-10 codes for all mental health and substance use disorder conditions in this report were based on codes for the VA risk-adjustment system called Nosos [8].

To develop corresponding ICD-10 diagnoses for the remaining conditions, we cross-walked the original ICD-9 codes for each condition to ICD-10 codes using general equivalency mapping (GEM) publicly available from the Centers for Disease Control and the Centers for Medicare and Medicaid Services, and these code mappings were reviewed by several clinicians to develop a final coding scheme. None of the condition definitions included ICD-10 codes referring to history of the relevant diagnosis since it was not clear that the diagnosis was applicable to the current year.

### Analysis

We identified a 20% random sample of all VA patients for a total of 1.0 million VA patients who used any VA inpatient or outpatient care in each year from FY2014–2016 and did not die during that time. We coded all their chronic conditions based on the presence of at least one diagnosis in each year from all VA inpatient and outpatient utilization records from the VA Medical SAS files which are national, administrative data containing all inpatient and outpatient encounters provided by the VA. These data include patient identifiers, location of care, procedures, and diagnoses. VA inpatient data included up to 14 diagnosis fields for FY14–15 and up to 26 fields for FY16 while outpatient data included up to 10 diagnosis fields for FY14–16, and we used all available diagnosis fields to identify the chronic condition codes.

For FY2014 we summarized the percent of patients who were identified with each condition using ICD-9 codes. For FY2015 we summarized the percent of patients who were identified with each condition a. who had the condition recorded in FY2014 and b. those who did not have the condition recorded in FY2015 using ICD-9 codes. We also summarized the percent of patients who had a diagnosis in FY2014 but not in FY2015. For FY2016 we summarized the percent of patients who were identified with each condition using ICD-10 codes a. who had the condition recorded in FY2015 and b. those who did not using ICD-9 codes and the percent of patients who had a diagnosis in FY2015 but not in FY2016.

In order to determine the magnitude in odds of a patient being diagnosed with a condition due to the change to ICD-10 coding, we conducted logistic regression models for each condition separately where we predicted having the condition with use of ICD-10 codes as a predictor (and ICD-9 was the reference group) and obtained the odds ratio (OR). Models adjusted standard errors for clustering within patient since patients had three observations for each year.

## Results

Condition prevalence rates were similar across all study years for most of the conditions, and there were large differences for several conditions (Table 1). The largest difference was the increase in Alzheimer’s disease from 0.4% in FY15 to 0.6% in FY16 with the odds of having Alzheimer’s disease 2.88 higher under ICD-10 compared to ICD-9. Other large differences in total condition rates occurred for an increase in heart failure (OR = 1.66; *P* < 0.001) from 4.6% in FY15 to 5.2% in FY16, an increase in spinal cord injury (OR = 2.05; *P* < 0.001) from 0.4% in FY15 to 0.5% in FY16, a decrease in HIV/AIDS (OR = 0.31; *P* < 0.001) from 0.55% in FY15 to 0.49% in FY16, and a decrease in arthritis (OR = 0.55; *P* < 0.001) from 20.0% in FY15 to 14.8% in FY16.

**Table 1:** Rates of Chronic Conditions Among VA Patients Using ICD-9 and ICD-10 codes, *N* = 1.0 million

Conditions	Dx in FY14	Dx in FY15		% Dx in FY14 but not FY15	Dx in FY16		% Dx in FY15 but not FY16	Total Rate FY14	Total Rate FY15	Total Rate FY16	Odds Ratio for ICD-10 Predicting Dx
		Also Dx in FY14	Newly Dx in FY15		Also Dx in FY15	Newly Dx in FY16					
Physical Health											
Acid Related Diseases	19.09%	12.47%	6.91%	6.61%	10.60%	6.86%	8.79%	19.1%	19.4%	17.5%	0.79
Alzheimer’s Disease	0.31%	0.21%	0.20%	0.10%	0.25%	0.37%	0.17%	0.3%	0.4%	0.6%	2.88
Arthritis	19.51%	12.24%	7.79%	7.26%	8.70%	6.11%	11.33%	19.5%	20.0%	14.8%	0.55
Asthma	3.81%	2.52%	1.33%	1.28%	1.99%	1.12%	1.85%	3.8%	3.8%	3.1%	0.60
Cancer (all types)	10.15%	7.30%	3.70%	2.85%	6.81%	3.84%	4.20%	10.2%	11.0%	10.6%	1.02†
Chronic Obstructive Pulmonary Disease	9.87%	7.57%	3.23%	2.30%	7.44%	3.38%	3.36%	9.9%	10.8%	10.8%	1.16
Colorectal Cancer	0.76%	0.52%	0.28%	0.24%	0.41%	0.25%	0.39%	0.8%	0.8%	0.7%	0.67
Diabetes	26.48%	24.63%	2.79%	1.85%	24.91%	2.98%	2.51%	26.5%	27.4%	27.9%	1.41
HIV/AIDS	0.54%	0.52%	0.03%	0.02%	0.47%	0.02%	0.08%	0.5%	0.5%	0.5%	0.31
Headache	6.22%	3.27%	3.16%	2.95%	2.76%	2.68%	3.66%	6.2%	6.4%	5.4%	0.75
Heart Failure	3.97%	2.87%	1.70%	1.10%	2.94%	2.27%	1.63%	4.0%	4.6%	5.2%	1.66
Hepatitis C	2.66%	2.14%	0.66%	0.52%	1.96%	0.58%	0.84%	2.7%	2.8%	2.5%	0.77
Hypertension	55.37%	48.46%	7.35%	6.91%	46.22%	7.93%	9.59%	55.4%	55.8%	54.1%	0.85
Ischemic Heart Disease	16.06%	12.88%	3.73%	3.18%	11.10%	3.65%	5.51%	16.1%	16.6%	14.8%	0.69
Lower Back Pain	24.27%	16.49%	9.00%	7.78%	14.84%	8.43%	10.65%	24.3%	25.5%	23.3%	0.84
Lung Cancer	0.47%	0.37%	0.26%	0.10%	0.33%	0.27%	0.30%	0.5%	0.6%	0.6%	1.20
Multiple Sclerosis	0.32%	0.29%	0.03%	0.03%	0.29%	0.04%	0.04%	0.3%	0.3%	0.3%	1.05†
Parkinson’s Disease	0.81%	0.69%	0.24%	0.12%	0.73%	0.26%	0.20%	0.8%	0.9%	1.0%	1.69
Peripheral Vascular Disease	4.72%	3.10%	2.10%	1.62%	2.79%	2.63%	2.41%	4.7%	5.2%	5.4%	1.21
Pneumonia	1.18%	0.22%	1.22%	0.96%	0.24%	1.37%	1.21%	1.2%	1.4%	1.6%	1.27
Prostate Cancer	4.48%	3.49%	1.22%	0.99%	3.04%	1.08%	1.67%	4.5%	4.7%	4.1%	0.70
Prostatic Hyperplasia	11.96%	7.71%	4.84%	4.25%	5.93%	4.47%	6.61%	12.0%	12.5%	10.4%	0.70
Renal Failure	6.49%	4.65%	2.79%	1.84%	4.54%	2.65%	2.90%	6.5%	7.4%	7.2%	1.09
Spinal Cord Injury	0.39%	0.34%	0.08%	0.05%	0.34%	0.14%	0.08%	0.4%	0.4%	0.5%	2.05
Stroke	3.71%	2.36%	1.69%	1.35%	1.95%	2.64%	2.11%	3.7%	4.1%	4.6%	1.39
Mental Health											
Dementia	1.69%	1.25%	0.87%	0.44%	1.33%	1.14%	0.79%	1.7%	2.1%	2.5%	1.86
Alcohol Dependence	4.05%	2.45%	1.57%	1.60%	1.57%	1.43%	2.46%	4.1%	4.0%	3.0%	0.56
Alcohol Abuse	3.76%	1.93%	1.96%	1.83%	1.37%	2.07%	2.52%	3.8%	3.9%	3.4%	0.84
Drug Dependence	4.49%	2.77%	1.88%	1.72%	2.09%	1.48%	2.56%	4.5%	4.7%	3.6%	0.60
Tobacco/Nicotine Dep	16.02%	10.94%	4.95%	5.08%	8.39%	3.98%	7.49%	16.0%	15.9%	12.4%	0.52
Schizophrenia	1.70%	1.50%	0.22%	0.20%	1.44%	0.26%	0.28%	1.7%	1.7%	1.7%	0.99†
Bipolar [Manic Depression]	2.65%	2.13%	0.58%	0.52%	1.95%	0.53%	0,75%	2.7%	2.7%	2.5%	0.74
Depression	20.97%	15.48%	6.08%	5.49%	13.08%	5.16%	8.48%	21.0%	21.6%	18.2%	0.64
PTSD	14.56%	12.17%	3.05%	2.39%	11.89%	2.77%	3.33%	14.6%	15.2%	14.7%	0.93

Dx = diagnosed with ICD-9 or ICD-10 codes

Patients were identified with each condition if they had at least one inpatient/outpatient encounter with one of the indicated diagnosis ICD-9 codes in FY2014–2015 and ICD-10 codes in FY2016

†All conditions had odds ratios with a *P*-value <0.001 with the exception of all cancers (*P* = 0.005), multiple sclerosis (*P* = 0.406), and schizophrenia (*P* = 0.675)

Additionally, there were large changes in several mental health and substance use conditions including a decrease in alcohol dependence (OR = 0.56; *P* < 0.001) from 4.0% in FY15 to 3.0% in FY16, a decrease in drug dependence (OR = 0.60; *P* < 0.001) from 4.7% in FY15 to 3.6% in FY16, and a decrease in tobacco/nicotine dependence (OR = 0.52; *P* < 0.001) from 15.9% in FY15 to 12.4% in FY16. All of the changes noted were associated with a greater than 20% change in the overall rate from FY15 to FY16.

For stroke and alcohol dependence, there was a large decrease in FY2016 in the number of patients who had the diagnosis in the current year and were also diagnosed in the previous year and a large increase in patients who were not diagnosed with these conditions in the current year but were in the year prior compared to the same statistics for the previous year. For depression in FY2016, there was a large increase in the percent of patients who did not have a diagnosis in the current year although they did have a depression diagnosis in the previous year, and this pattern deviated from the year before.

## Discussion

Overall, we were able to map ICD-9 and ICD-10 diagnosis codes to obtain condition prevalence rates that were similar before and after the transition to ICD-10 for most of the conditions we examined although there were some differences across conditions. We did not find any consistent decrease or increase in the rates of most conditions after the transition. We were unable to determine the reasons for changes in condition rates from FY15 to FY16 although the transition to ICD-10 may account for some of these differences. For all of the conditions, some year-to-year variation may be due to treatment patterns such as patients not having the condition treated during that year or not being recorded in utilization data. A portion of patients who are newly diagnosed in each year may not have developed the condition until the current year, or else the condition was not previously recorded. Since we did not find any consistent decrease in chronic condition rates after FY2015, there does not appear to be systematic undercoding of diagnosis codes after the transition to ICD-10. This is consistent with research using Canadian hospital discharge data that found that ICD-9 and ICD-10 coding systems performed similarly in predicting mortality of chronically ill patients [9].

It is unknown why there were large changes in some conditions, especially decreases in rates of mental health and substance use disorder conditions after transition to ICD-10 and whether any of the decreases were related to undercoding of these conditions. There appeared to be a more restrictive cohort for many of these conditions under FY16. Mental health conditions may not be the primary reason for some patients receiving care, so it is possible that providers may be less likely to enter diagnosis codes for secondary conditions in ICD-10. Mental health conditions were previously identified as a clinical area with especially convoluted mappings from ICD-9 to ICD-10, so the learning curve may be higher for these conditions. It is also possible that more specific categories under ICD-10 led to greater accuracy in recording which patients had mental health conditions, which also resulted in lower rates. Future work should examine these issues in greater detail to ensure conditions are accurately and comprehensively recorded in the future.

### Limitations

There were several limitations in our analysis. While we examined changes in rates of our study conditions, it is possible that there may have been differences in rate changes between sub-types of conditions such as mild, moderate, or severe asthma. Another limitation of our data involves subgroups of patients (e.g. older, male) who have higher rates of certain conditions; subgroups of patients who are more likely to be diagnosed with certain conditions might be more affected by coding changes, so further examination of this question may be needed. Lastly, the maximum number of diagnosis fields for inpatient utilization records changed from 14 in FY14–15 to 26 fields in FY16 which allowed for recording of more diagnoses which can potentially affect condition rates; however, we found that the mean number of diagnoses entered per encounter record was similar across all study years (1.5 in FY14, 1.6 in FY15, and 1.4 in FY16). Therefore, change in number of diagnosis fields did not appear to be a factor in changes in condition rates in our study population.

## Conclusion

While population rates of common chronic conditions were generally stable over time, there may be differences in how individual patients were identified with conditions such as mental health and substance abuse and several other conditions under ICD-10, so health care systems and health services researchers should take these differences into account in the measurement of chronic conditions in patient populations over time.

## Abbreviations

CMS: Centers for Medicare & Medicaid Services
Dx: Diagnosed
FY: Fiscal years
GEM: General equivalency mapping
HIPAA: Health Insurance Portability and Accountability Act
ICD-10-CM: International Classification of Diseases and Related Health Problems, 10th revision, Clinical Modification
ICD-9-CM: International Classification of Diseases and Related Health Problems, 9th revision, Clinical Modification
OR: Odds ratio
SAS: Statistical Analysis System
VA: Veterans Affairs
